# Defective Fluid Secretion from Submucosal Glands of Nasal Turbinates from CFTR^-/-^ and CFTR^ΔF508/ΔF508^ Pigs

**DOI:** 10.1371/journal.pone.0024424

**Published:** 2011-08-31

**Authors:** Hyung-Ju Cho, Nam Soo Joo, Jeffrey J. Wine

**Affiliations:** Cystic Fibrosis Research Laboratory, Psychology Department, Stanford University, Stanford, California, United States of America; Johns Hopkins School of Medicine, United States of America

## Abstract

**Background:**

Cystic fibrosis (CF), caused by reduced CFTR function, includes severe sinonasal disease which may predispose to lung disease. Newly developed CF pigs provide models to study the onset of CF pathophysiology. We asked if glands from pig nasal turbinates have secretory responses similar to those of tracheal glands and if CF nasal glands show reduced fluid secretion.

**Methodology/Principal Findings:**

Unexpectedly, we found that nasal glands differed from tracheal glands in five ways, being smaller, more numerous (density per airway surface area), more sensitive to carbachol, more sensitive to forskolin, and nonresponsive to Substance P (a potent agonist for pig tracheal glands). Nasal gland fluid secretion from newborn piglets (12 CF and 12 controls) in response to agonists was measured using digital imaging of mucus bubbles formed under oil. Secretion rates were significantly reduced in all conditions tested. Fluid secretory rates (Controls vs. CF, in pl/min/gland) were as follows: 3 µM forskolin: 9.2±2.2 vs. 0.6±0.3; 1 µM carbachol: 143.5±35.5 vs. 52.2±10.3; 3 µM forskolin + 0.1 µM carbachol: 25.8±5.8 vs. CF 4.5±0.9. We also compared CF^ΔF508/ΔF508^ with CFTR^-/-^ piglets and found significantly greater forskolin-stimulated secretion rates in the ΔF508 vs. the null piglets (1.4±0.8, n = 4 vs. 0.2±0.1, n = 7). An unexpected age effect was also discovered: the ratio of secretion to 3 µM forskolin vs. 1 µM carbachol was ∼4 times greater in adult than in neonatal nasal glands.

**Conclusions/Significance:**

These findings reveal differences between nasal and tracheal glands, show defective fluid secretion in nasal glands of CF pigs, reveal some spared function in the ΔF508 vs. null piglets, and show unexpected age-dependent differences. Reduced nasal gland fluid secretion may predispose to sinonasal and lung infections.

## Introduction

Cystic fibrosis (CF) occurs when CFTR anion channels, which are especially important in the functioning of ion transporting epithelia, are missing or defective. An elevated transepithelial difference across the nasal epithelium was the first electrophysiological signature of an ion transport defect in CF [1]. That finding led to extensive research on electrolyte transport defects in CF respiratory epithelia and glands. One goal of that research is to understand how altered epithelial ion and fluid transport predisposes the respiratory system to chronic bacterial infections, which are presently the most significant clinical feature of CF. Almost all CF patients have upper airway abnormalities, and ∼50% have chronic rhinosinusitis (CRS) and/or nasal polyps [2]. In the non-CF population, CFTR mutations on one chromosome predispose to CRS [3], sometimes including bacterial biofilms on the nasal mucosa [4]. It is hypothesized that sinonasal bacterial infections can seed the lower airways, based on evidence for earlier onset of upper airway infections and concordance between sinonasal and lower airway pathogens [5], [6], [7], [8], [9], [10], [11], [12], [13]. Nasal epithelium is easier to access than the lower airways, for which it often serves as a surrogate to assess CFTR function in humans, *in vivo* [14], [15], [16], or in tissue culture [17], [18], [19].

In the trachea and bronchi submucosal glands express CFTR [20], and are functionally important in antimicrobial defense; ferret tracheal xenografts lacking submucosal glands produce less lysozyme and are more prone to infection than xenografts with glands [21]. Mucus accumulation and obstruction of submucosal glands, secondary to decreased fluid secretion, has been suggested to be a contributing factor to the pathophysiology of patients with CF [22], [23], [24], [25], and in CF human bronchi, the submucosal glands lack or have reduced fluid secretion in response to multiple agonists [26], [27], [28], [29]. Nasal glands have only rarely been studied directly in CF subjects, but in an important exception, gland secretion in response to pilocarpine was reduced and more acidic in nasal biopsy tissue from relatively healthy CF subjects [30]. However, the links between the ion transport dysfunctions of human CF airway glands and surface epithelia and CF lung disease remain obscure.

Animal models that mimic human CF are a potential solution to this research bottleneck. Among animals, pig airways share many features with human airways [31] and CF pigs, both CFTR^-/-^ [32] and CFTR^ΔF508/ΔF508^ [33] have now been generated and shown to share many features of CF organ-level disease [34], [35], [36], [37], including a defect in tracheal host defense against bacterial infections [37], and defective tracheal gland secretion [38]. Because of severe meconium ileus, most CF piglets are euthanized shortly after birth. However, a few pigs that survived up to 6 months after intestinal bypass surgery had airways infected with multiple organisms and displayed mucus accumulation and airway remodeling [37]. Rhinosinusitis in the CF pig has not yet been reported, but its nasal epithelium exhibited defective chloride transport [32].

We discovered several novel features of nasal submucosal glands and have also shown differences between control and CF glands, and between glands having CFTR^-/-^ and CFTR^ΔF508/ΔF508^ genotypes. We also discovered marked, age-related differences in the relative magnitude of forskolin and carbachol-stimulated secretion.

## Results

### Size, density and secretory capacity of nasal vs. tracheal glands as a function of age

**Figure 1:**
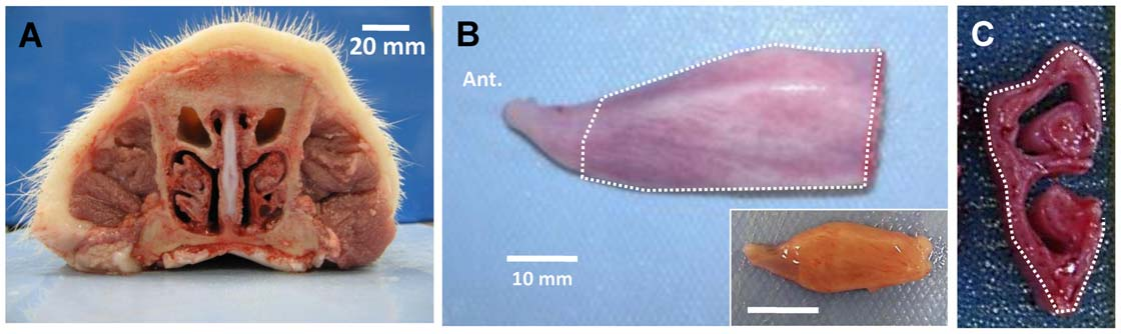
Gross anatomy of pig nasal cavity and turbinate. **A**, Cross-section of adult pig nose observed from caudal side. **B**, Ventral nasal turbinate from right side of nasal cavity of adult pig, observed from medial side. Inset shows turbinate from a neonatal piglet. **C**, Cross-sectioned turbinate showing superior and inferior scrolls. Dotted lines in **B** and **C** indicate convex medial side of ventral nasal turbinate where most submucosal glands are located and where mucus bubbles were observed. Scale bars: 20 mm (**A**); 10 mm (**B**).

Each nasal turbinate is attached to the lateral wall of the nasal cavities and comprises superior and inferior scrolls (Figure 1A, C). The extent of the turbinate mucosa used for measuring mucus secretion from glands is indicated with the dotted lines in Figure 1B, C.

**Figure 2:**
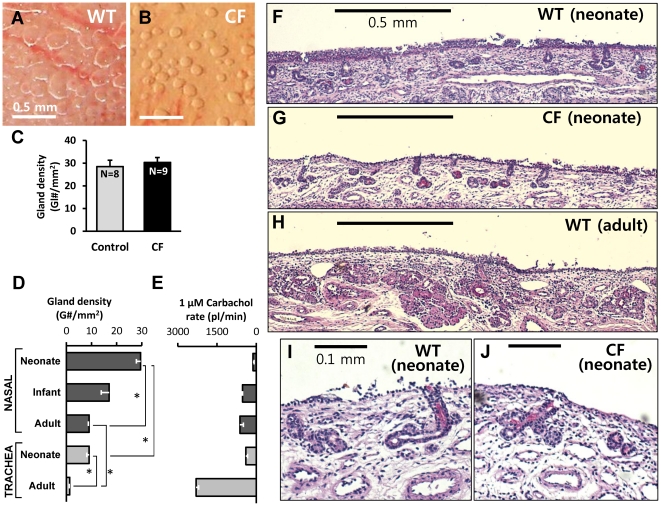
Overview of submucosal glands from pig nasal turbinates. **A and B**, Examples of mucous bubbles formed under oil on the surface of nasal turbinates after 5 min stimulation with 1 µM carbachol of WT (**A**) and CF (**B**) piglets. This type of measurement was used to determine the number of functional nasal glands per unit area of surface shown in (**C**). **C,** The average number of functional nasal glands per mm^2^ of surface did not differ between control and CF piglets; values were 29±3 (8 WT + 2 Hz piglets) vs. 30±2 in 9 CF piglets (*P* = 0.58, n.s.). **D**, Gland density as a function of age and location. Nasal gland density is significantly greater than tracheal gland density (*P*<0.0001), and neonatal gland density is greater than adult gland density (*P*<0.0001). Values per mm^2^ were: neonatal turbinate: 29.4; neonatal trachea: 9.01; adult turbinate: 9.05; adult trachea: 1.33. **E**, Single gland secretion rates were inversely related to gland density, being lowest in neonatal nasal glands and highest in adult tracheal glands. **F**–**J**, Histology of nasal submucosal glands from WT neonate (**F, I**), CF neonate (**G, J**), and WT adult (**H**) pig (hematoxylin and eosin with periodic acid-Schiff stain, x40 (**F**–**H**), x100 (**I, J**)). Scale bars: 0.5 mm (**A, B, F, G, H**); 0.1 mm (**I, J**).

When the turbinate mucosa is covered with an oil layer and stimulated, the secretions from the turbinate submucosal glands form dense arrays of mucus bubbles. In neonatal piglets, the number and density of CF and WT glands did not appear to differ based on counts of the mucus bubbles per unit area of turbinate and submucosal gland histology (Figure 2A, B, F, G).

We compared the density of submucosal glands in the nasal turbinates with those in the anterior trachea, and compared neonates with adults. In the areas of highest gland density, measured on the area indicated in Figure 1B, C, glands were ∼3-fold more abundant than in the anterior trachea (Figure 2D). Gland density decreased with age both in nasal turbinates and in trachea, consistent with the number of glands being fixed near the time of birth [39], so that the same number of glands serves a larger surface area (Figure 2D). The decline was greater in the trachea, where the density in adults was ∼15% of neonatal piglets, compared with nasal turbinates, where the density in adults was ∼33% of neonatal piglets. Single gland secretion rates (in response to 1 µM carbachol) showed an opposite trend (Figure 2E). In tracheal glands, secretion rates of adults are roughly 5-fold greater than rates of 1-day old piglets, while in nasal glands secretion rates of adults are roughly 4-fold greater than rates of 1-day old piglets.

Although the number and volume of nasal glands appeared to be unchanged by visual inspection of 3 samples of WT and CF turbinates (Figure 2I, J, Figure S1), we did not quantify gland volumes with morphometric analysis. Analysis of sectioned tracheas was used by Meyerholz *et al.* to show that tracheal gland volume of CF piglets was reduced by 31%–37% [36], and a similar reduction might be present in the turbinate glands, although the CF pig nasal cavity does not display the gross morphological changes that are obvious upon visual inspection of the trachea. Therefore some of the reduced secretory responsiveness of the CF glands might result from decreased gland volume (see discussion).

### CF piglets show reduced nasal gland fluid secretion in response to forskolin

**Figure 3:**
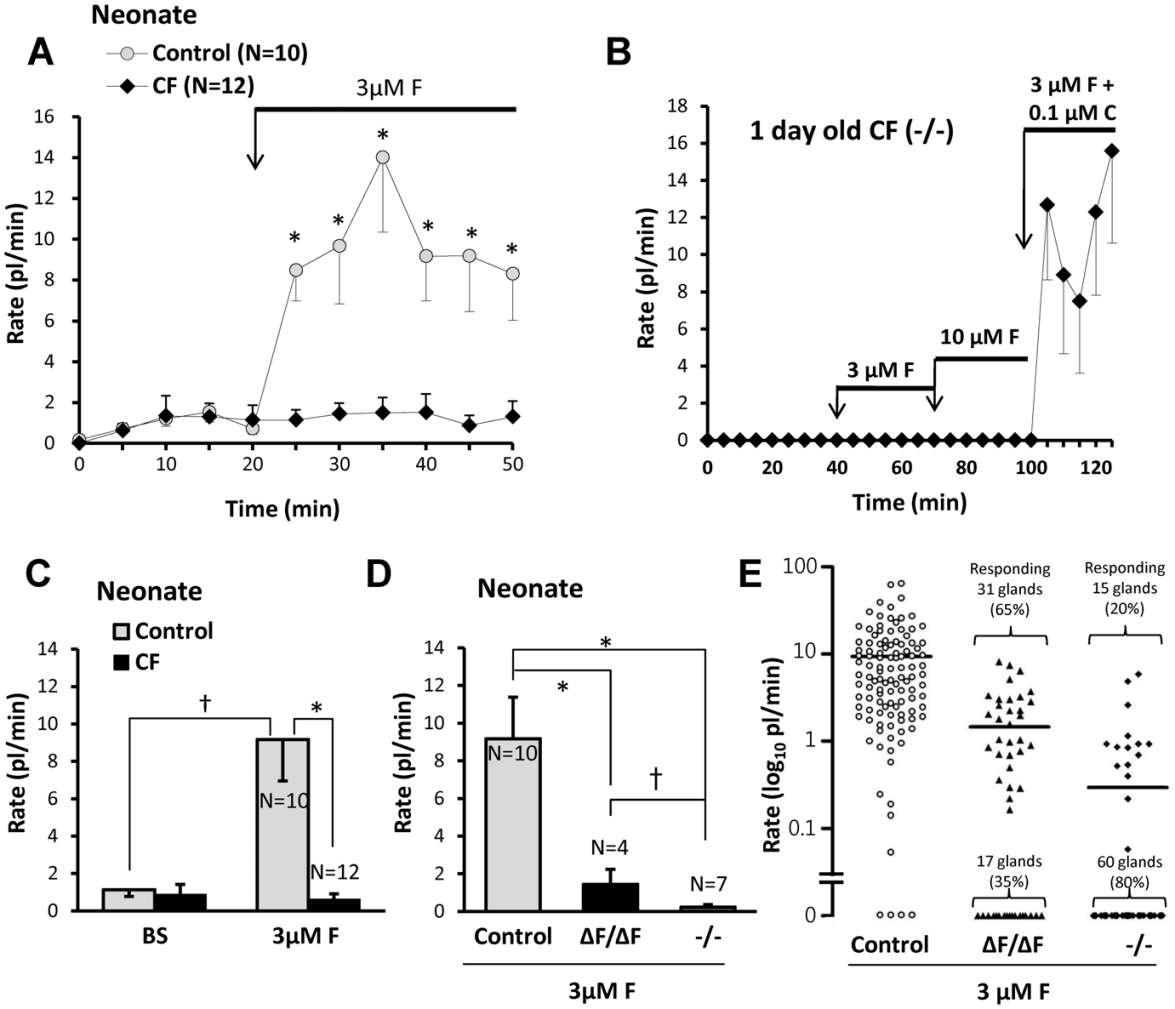
Defective forskolin-stimulated fluid secretion from CF piglet nasal glands. **A**, Plots of averaged nasal gland secretion rates from 8 control and 9 CF piglets stimulated with 3 µM forskolin. The response for each piglet was the average of from 8 to 17 glands. Significantly more fluid was secreted at the first 5 min time point and at each subsequent point (t-test, * indicates *P*<0.05). **B**, Response to small carbachol stimulus proves CF nasal glands are viable. Average secretion rates of 9 nasal glands from a 1 day old CF (-/-) piglet as a function of time and stimulation with forskolin (3 and 10 µM) and carbachol (0.1 µM). **C**, Summary data for 10 control and 12 CF piglets (* indicates t-test *P*<0.003, † indicates paired t-test *P*<0.006). Rates were averaged over the entire 30 min period of stimulation and the basal rate was subtracted from the forskolin-stimulated rates. If rates are determined for only the last 20 min of stimulation (sustained rates) the rate is ∼10% higher for forskolin and ∼20% less for carbachol. The response of CF glands to 3 µM forskolin did not differ significantly from zero. **D**, Mean response rates to 3 µM forskolin plotted separately for each genotype. (* indicates t-test *P*<0.006, † indicates Mann-Whitney test *P*<0.03). **E**, Response rates for all glands tested, plotted separately for genotype; horizontal bars indicate means.

Tracheal submucosal glands of CF humans, pigs, mice and ferrets have profound reductions in their secretory responses to forskolin [26], [38], [40], [41]. To determine if CF piglet nasal glands were similarly impaired, we measured their responses to forskolin stimulation (Figure 3). Average basal (unstimulated) secretion rates were <2 pl/min/gl in all genotypes (n = 22). In WT nasal glands forskolin significantly increased the secretion rate ∼7-fold within 5 min (*P*<0.002, n = 22). Secretion was sustained for at least 30 min (Figure 3A, gray symbols). In contrast, CF gland secretion rates did not change significantly after forskolin (Figure 3A, black symbols). CF nasal gland viability was established by showing responsiveness to carbachol after the glands failed to respond to two concentrations of forskolin (Figure 3B). Mean data for forskolin experiments with 10 control piglets (8 WT + 2 Hz) and 12 CF piglets are shown in Figure 3C. The forskolin-stimulated secretion rate in CF piglets (0.6±0.3 pl/min) did not differ significantly from the basal rate (*P* = 0.66) and was significantly reduced relative to control piglets (9.2±2.2 pl/min; *P*<0.0003).

### Comparison of CF^ΔF508/ΔF508^ and CF^-/-^ piglet responses to forskolin

We compared forskolin-stimulated nasal gland fluid secretion from CF^ΔF508/ΔF508^ vs. CF^-/-^ genotypes (Figure 3D, E). The mean secretion rate for the CF^ΔF508/ΔF508^ piglets was 1.4±0.8 picoliters/min/gland, n = 4, 48 glands vs. 0.2±0.1 for CF^-/-^ piglets (n = 7, 75 glands, *P*<0.03). Most glands from either phenotype did not respond to forskolin. To compare the two genotypes further, we plotted results for 112 individual control glands and 123 CF glands: 75 glands from CF^-/-^ and 48 from CF^ΔF508/ΔF508^ pigs. Overall, 46/123 (37%) of CF glands responded to forskolin, with 31/48 (65%) of the CF^ΔF508/ΔF508^ glands responding vs. 15/75 (20%) were from the CF^-/-^ genotype. The average secretion rate of the responding CF^ΔF508/ΔF508^ glands was 1.6 fold higher than that of the responding CF^-/-^ glands (Figure 3E).

### Comparisons of tracheal vs. nasal glands and neonate nasal vs. adult nasal glands revealed differential responsiveness to agonists

**Figure 4:**
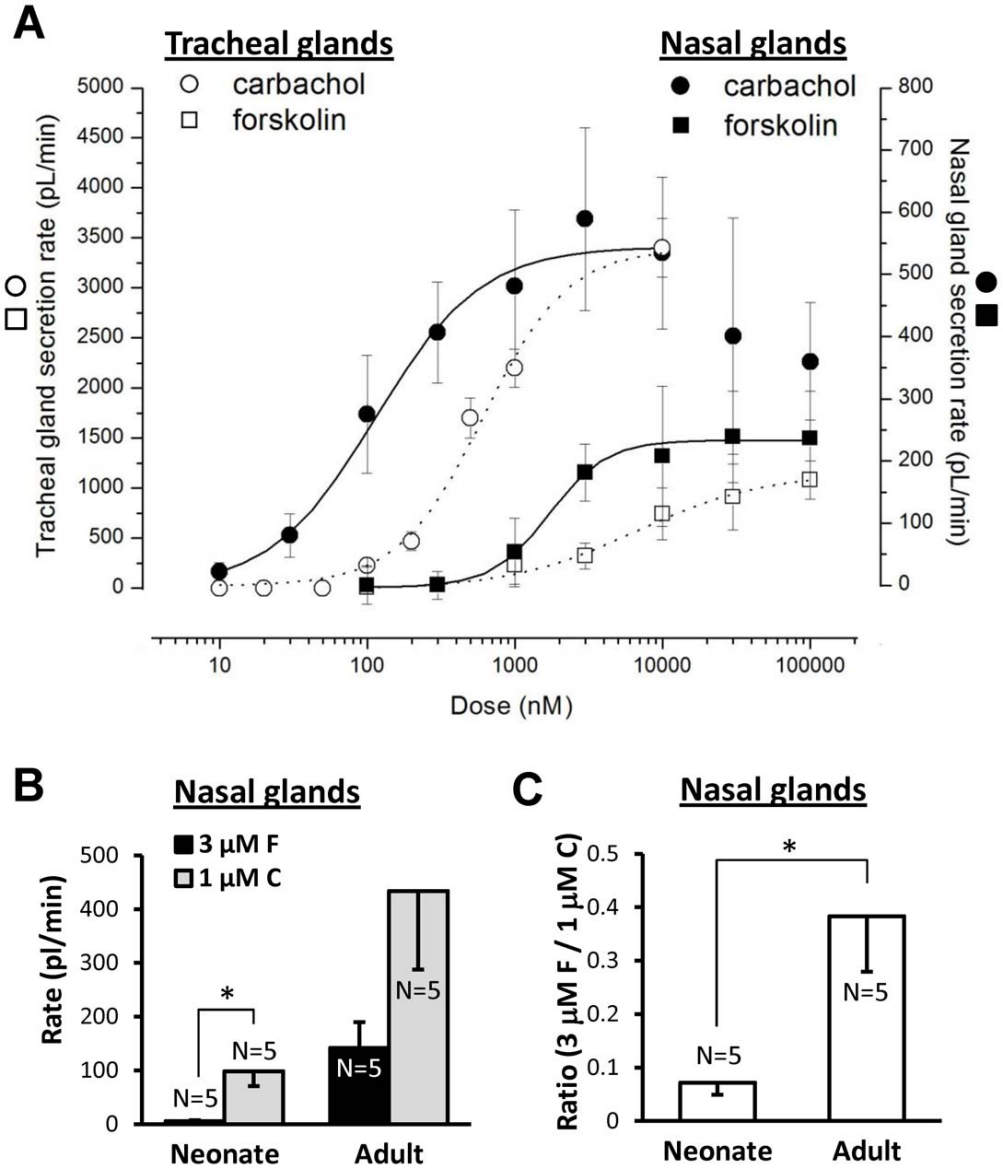
Agonist sensitivities. **A**, Nasal glands are more sensitive than tracheal glands to both carbachol and forskolin. Dose-response plots for carbachol (circles) and forskolin (square) in nasal glands (solid line, closed symbols) and tracheal glands (dotted line, open symbols) of adult pigs. Tracheal gland responses are plotted on the left Y axis and nasal gland responses on the right Y axis; the two Y axes were adjusted to give overlapping maximal response rates to carbachol. The carbachol dose-response for tracheal glands (dotted line, open circles) is from Choi *et al.*, 2007 [27]. Each point represents average data from 33–80 glands in 3–7 pigs; doses were increased sequentially except for concentrations ≥3 µM forskolin and ≥1 µM carbachol, where single doses were tested in each experiment to avoid desensitization and merging of bubbles. **B**, nasal gland secretion rates of neonates and adults to carbachol and forskolin (*indicates paired t-test, *P*<0.02). **C**, The *ratio* of secretion rates to forskolin vs. carbachol was significantly greater in adult than in neonatal pigs (t-test, * indicates *P*<0.02).

We established dose-response relationships to carbachol and forskolin in nasal glands from adult pigs for comparison with dose-response relationships for adult pig tracheal glands obtained previously by Choi *et al.* [27]. Both agonists showed increased potency for nasal glands: the EC_50_ for carbachol was ∼20% of that observed previously for tracheal glands (114±18 nM vs. 590±16 nM), and the EC_50_ for forskolin was ∼ 25% of that observed previously for tracheal glands (1691±135 nM vs. 6635±1836 nM), Figure 4A.

In adult pig glands carbachol's potency and efficacy are greater than forskolin's (Figure 4A). Unexpectedly, we discovered a marked, age-dependent difference in the relative efficacy of carbachol and forskolin in nasal glands of neonate vs. adult pigs. In neonatal piglet nasal glands, 1 µM carbachol stimulated a mean secretion rate ∼18-fold greater (*P*<0.02) than that produced by 3 µM forskolin, whereas in adult pigs it was about 3-fold greater (Figure 4B). When the forskolin/carbachol ratio was determined within individual neonatal and adult pigs, the ratio was ∼5-fold greater in adults (*P*<0.03; Figure 4C).

### Carbachol-stimulated fluid secretion from nasal glands is significantly reduced in CF piglets

**Figure 5:**
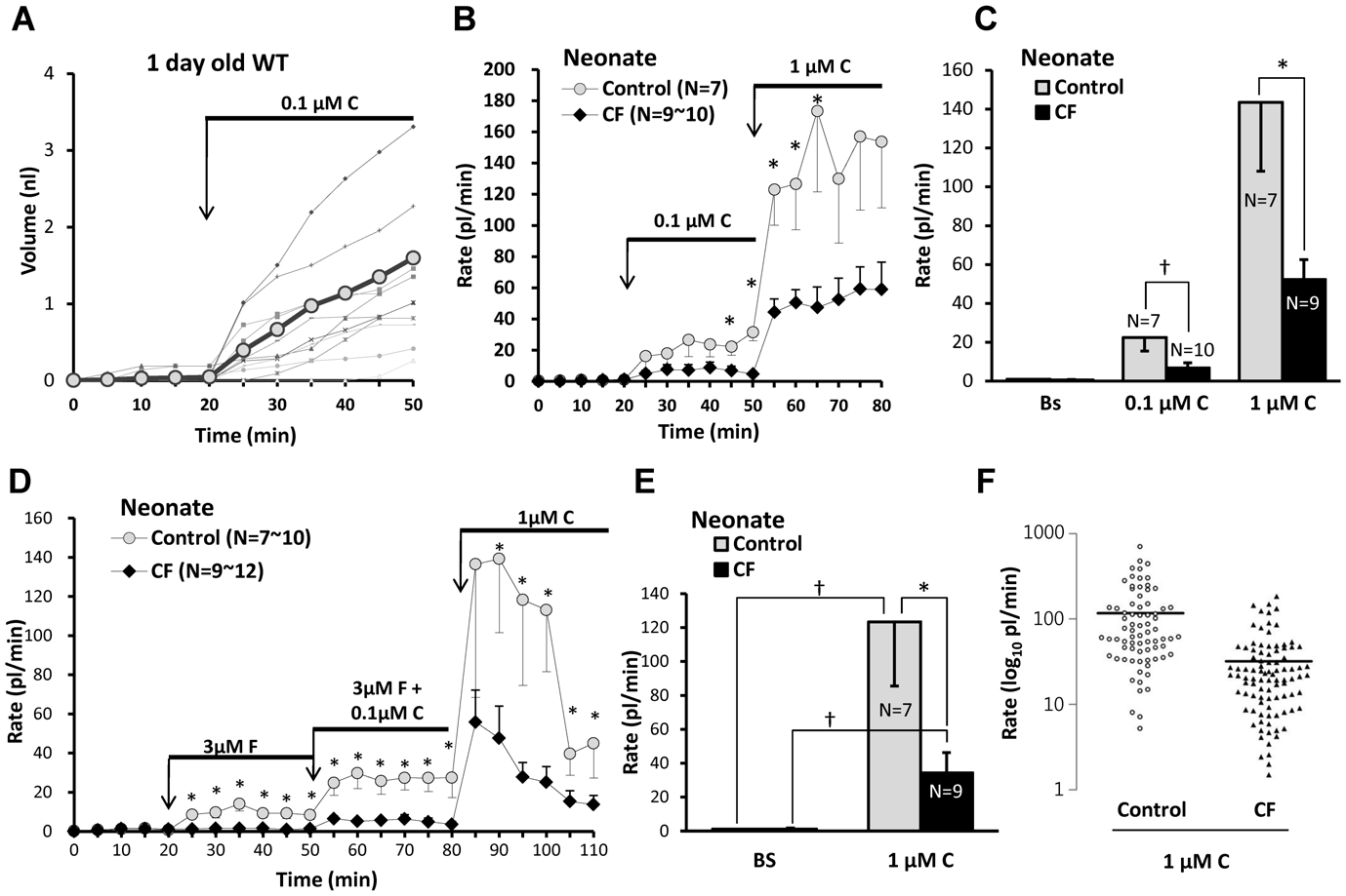
Carbachol-stimulated secretion from CF nasal submucosal glands is reduced. All data are from a one day old WT pig. **A**, A representative experiment plotting mucus volume as a function of time and stimulation with 0.1 µM carbachol for 12 nasal glands from a newborn WT piglet. Thicker plot line and gray circle symbols indicate averaged volume. **B**, Average secretion rates as a function of time and stimulation for 7 control and 9–10 CF piglets stimulated with 0.1 µM and then 1 µM carbachol. **C**, Summary data: 1 µM carbachol caused a significant increase is secretion from nasal glands of both control and CF piglets; the response in CF piglets was significantly smaller than in control piglets († indicates Mann-Whitney test, *P*<0.03, * indicates t-test, *P*<0.04). Secretion rates to 0.1 µM carbachol were (in pl/min/gland) control: 22.4±7.0 (n = 7) and CF: 6.7±2.6 (n = 10). Rates to 1 µM carbachol were control: 143.5±35.5 (n = 7) and CF was 52.2±10.3 (n = 9). **D**, Average nasal gland secretion rates as a function of time and stimulation for 10 control and 9–12 CF piglets stimulated sequentially with 3 µM forskolin, 3 µM Forskolin + 0.1 µM carbachol, and 1 µM carbachol. **E**, Mean basal and 1 µM carbachol- stimulated secretory rates from experiment shown in (**D**). Bars represent the average secretion rates for the 30 min period following addition of carbachol, the difference between this graph and (**C**) is that carbachol stimulation here directly followed forskolin stimulation after brief washout (* indicates control vs. CF, t-test *P*<0.05, † indicates paired t-test *P*<0.03). **F**, Distribution of secretory rates for all glands in response to 1 µM carbachol.

To compare responses to carbachol in WT and CF nasal glands more directly, we augmented our standard protocol by testing carbachol alone at two concentrations. This avoided residual effects from forskolin, which precedes carbachol in our standard stimulation protocol. In WT piglets (Figure 5A–C), 0.1 µM carbachol increased secretion ∼39-fold over basal rates (n = 7, 84 glands, *P*<0.02). Responses of WT nasal glands to 0.1 and 1 µM carbachol were 3.3 and 2.7 fold larger than corresponding CF responses when comparing secretion rates over the entire 30 min period of carbachol-stimulated secretion, (0.1 µM: n = 7, 10 *P*<0.03; 1 µM: n = 7, 9, *P*<0.02). Secretion to carbachol has a large, transient component. When considering just the 15 min period post-stimulation with 1 µM carbachol, the control responses were also significantly larger than the CF responses (*P*<0.04; n = 7, 9). When 1 µM carbachol was given at the end of the standard stimulation protocol (Figure 5D), it stimulated nasal gland secretory rates that were 3.6-fold greater in WT vs. CF piglets. (Figure 5E, *P*<0.02). The distribution of individual gland responses for carbachol-stimulated secretion in the standard protocol is shown in Figure 5F.

### Nasal glands of neonatal piglets did not show synergy between forskolin and carbachol in the standard protocol

In tracheal submucosal glands of humans [27], [28], ferrets [42], and neonatal pigs [38], combining low levels of [cAMP]_i_ and [Ca^2+^]_i_ elevating agonists produces fluid secretion that is greater than the expected additive effect. This synergistic secretion is lost in CF glands of humans [27], [28] and pigs [38]. We included a test for synergy in piglet nasal glands using the same protocol that worked for piglet tracheal glands. However, as indicated in Methods, the concentrations of carbachol and forskolin in the standard protocol were non-optimal for demonstrating synergy. Nasal glands are more sensitive than tracheal glands to carbachol and forskolin, so the 0.1 µM dose of carbachol produced significant secretion on its own (Figure 5A), as did the 3 µM concentration of forskolin (Figure 2A). When combined, their effects were additive, not synergistic, unlike the results in day-old pig tracheal glands [38]. Further experiments will be required to verify the apparent lack of synergy in the nasal turbinate glands.

### Minimal secretory response of WT piglet nasal glands to Substance P

**Figure 6:**
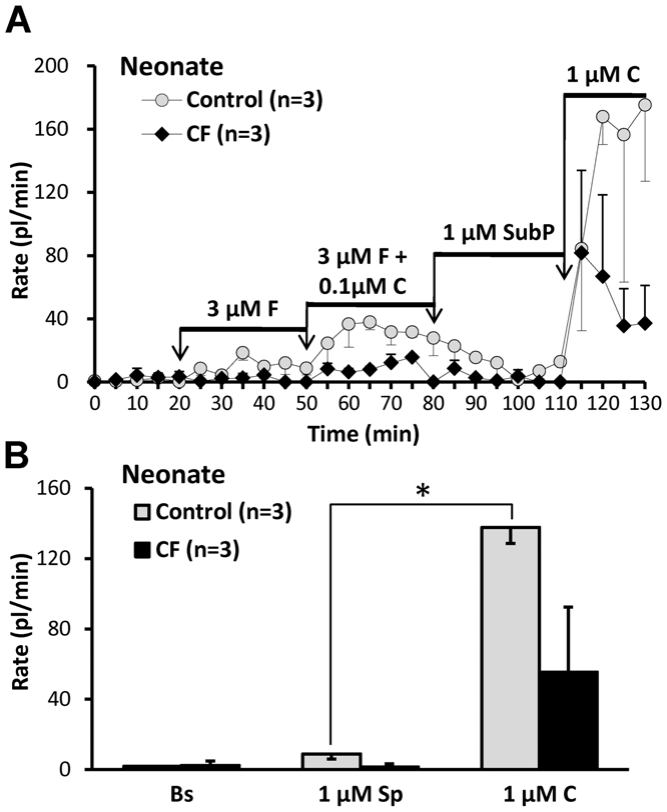
Nasal glands of newborn piglets show minimal secretory response to SubP. **A**, Averaged secretion rates as a function of time and stimulation from one day old WT and CF piglets (n = 3 each). Note especially the lack of response to SubP in both groups. **B**, Data from (**A**), replotted to show mean basal rates and rates to SubP and carbachol. Mean rates for control (in pl/min/gland) were: basal: 1.8±1.3; SubP: 8.8±2.8; carbachol: 137.7±9.0, (* indicates *P*<0.0002, n = 3, paired t-test, for difference between responses to SubP and carbachol. Mean rates for CF (in pl/min/gland) were: basal: 2.3±2.5; SubP: 1.5±1.8; carbachol: 55.4±37.0, (n = 3, paired t-test, *P* = 0.228).

**Figure 7:**
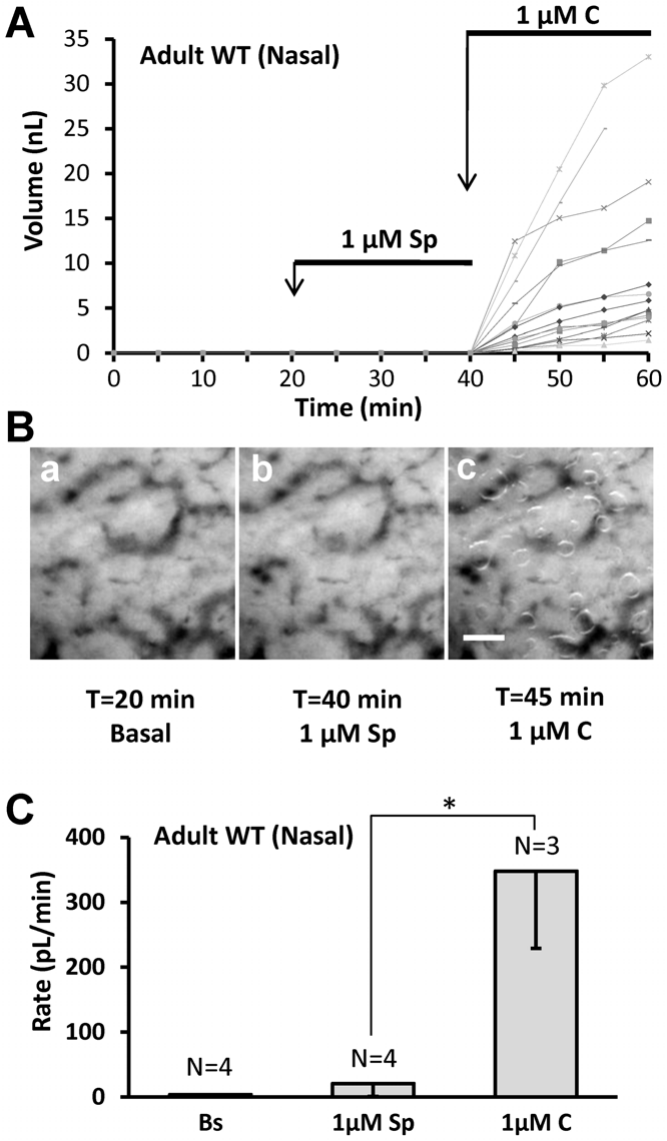
Nasal glands of adult WT pigs show minimal secretory response to SubP. **A**, Adult pig responses to SubP. Graph of mucus volume as a function of time and stimulation for 12 glands in fresh tissue from an adult pig. **B**, Images of mucus bubbles secreted under oil from nasal turbinate mucosa of an adult pig after stimulation with SubP for 20 min followed by carbachol for 5 min. **C**, Summary data for 3–4 pigs; the responses to SubP did not differ significantly from basal (paired t-test, *P* = 0.375) and were significantly less than the response to carbachol (t-test, *P*<0.02).

The tachykinin Substance P (SubP) stimulates tracheal gland secretion in humans [28], and pigs [43], but is a much better agonist for pig glands. For tracheal glands SubP is ∼10 times more efficacious in pigs than in humans [28]; in pigs SubP is more potent than acetylcholine and at least as efficacious [43]. We applied SubP to neonatal WT piglet nasal glands by modifying the standard protocol so that SubP (1 µM) was added instead of 1 µM carbachol following the 3 µM forskolin + carbachol 0.1 µM period. Surprisingly, secretion rates dropped to near basal levels in the presence of SubP after washout of forskolin + carbachol (Figure 6A). Figure 6B compares the average secretion rates to 1 µM SubP and 1 µM carbachol in 3 one-day old WT piglets; the secretion rate to carbachol was significantly (∼16-fold) greater than to SubP (*P*<0.004, paired t-test). The low responsiveness to SubP could be an intrinsic feature of nasal glands, or could have resulted from the young age of the piglets or from loss of responsiveness during overnight shipping. Therefore we tested nasal glands from four adult pigs obtained less than 2 hours after euthanasia and confirmed that the response to SubP was only ∼6% of the response to carbachol (Figure 7).

### Summary of gland secretory responses in nasal and tracheal glands of control and CF neonatal piglets

**Figure 8:**
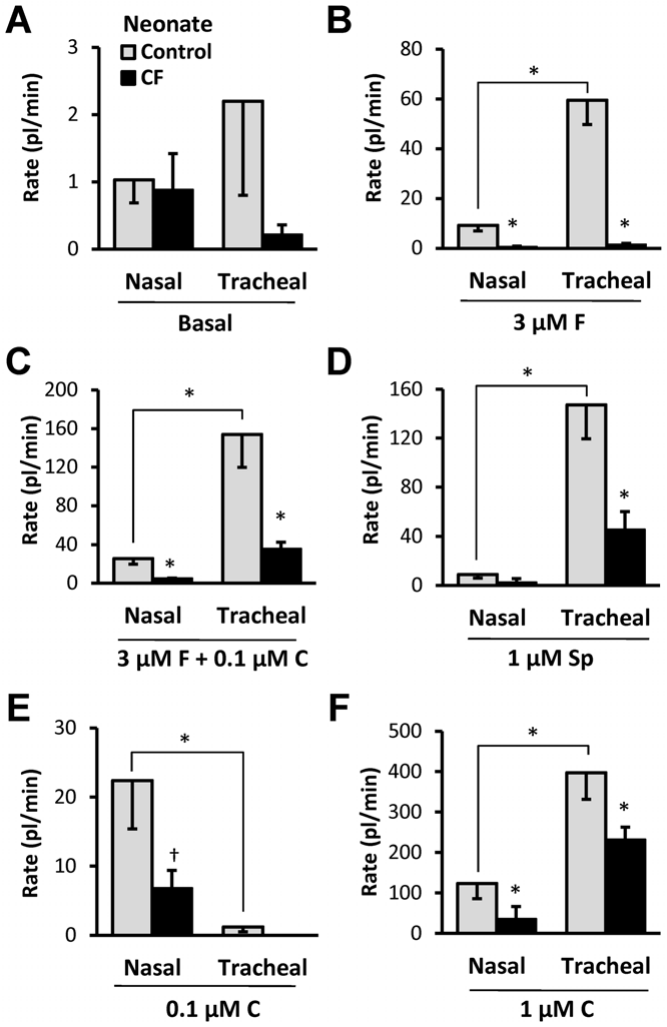
Summary of secretory responses comparing control vs. CF and nasal vs. tracheal glands in neonatal piglets. Data for tracheal responses taken from Joo *et al.*, [38]. **A**–**F**, Each graph shows control (open bars) and CF (closed bars) secretory rates for nasal (left) or tracheal (right) glands in response to the agents shown. * indicates t-test *P*<0.05 for either WT vs. CF or nasal vs. tracheal. † indicates Mann-Whitney test *P*<0.02. See text.

In Figure 8, secretion rates are shown for 6 stimulus conditions (A-F) with each bar representing one of 4 types of gland: nasal and tracheal from CF and control newborn piglets. Data for tracheal responses were taken from Joo et al. [38]. Responses to all agonists were significantly reduced in CF piglets except for SubP in nasal glands (not testable because no response in WT, and carbachol in tracheal glands (Figure 8B–D, F, *P*<0.05, t-test). WT nasal glands showed significantly smaller secretion rates than tracheal glands to all agonists except to 0.1 µM carbachol (Figure 8E), where the greater response of the nasal gland is thought to result from the higher potency to carbachol in nasal glands as shown in Figure 4A.

## Discussion

### CF vs. control glands

Nasal turbinate submucosal glands of one-day old CF piglets display a profound decrease in stimulated fluid secretion. Secretion to forskolin was absent, and secretion in response to carbachol alone or in combination with forskolin was reduced (Figure 2, 4, 5). Thus nasal glands, like tracheal-bronchial glands in other species, require CFTR for proper secretory function [25], [26], [27], [29], [30], [38], [40], [41], [44], [45], [46], [47], [48]. These results occurred prior to infection or inflammation. Decreased secretion was observed in both -/- and ΔF508/ΔF508 genotypes, with partial sparing in the ΔF508 genotype (Figure 3D, E). Partial sparing of forskolin-mediated secretion in the ΔF508 phenotype is consistent with evidence that porcine CFTR-ΔF508, expressed in various cell lines or human epithelia, confers more residual function than human CFTR-ΔF508 [49]. However, the amount of secretion observed is not sufficient to preclude the development of a CF phenotype, because *CFTR* ^ΔF508/ΔF508^ pigs develop a phenotype like that of CFTR^-/-^ pigs and humans with CF [33]. The occasional, very small secretory response to forskolin in CF^-/-^ nasal glands (Figure 3E) must occur via a non-CFTR pathway—perhaps via a forskolin elevation of [Ca^2+^]_i_ [50] that is on occasion sufficient to weakly activate CaCC.

The response to carbachol was also decreased in CF piglets. We have not yet measured the volumes of CF nasal glands with sufficient accuracy to eliminate an interpretation that part of the diminished response to carbachol reflects smaller gland volume. However, inspection of sections stained with H&E did not reveal obvious differences between CF and WT glands. Because similar reductions in secretory rates have been observed in CF human [30], ferret [40], and piglet glands [38], as well as in pig glands treated with CFTR_inh_-172 [48], we think these differences reflect the loss of CFTR-mediated fluid secretion.

### Nasal vs. tracheal glands

We also documented functional differences between nasal and tracheal glands. The sinonasal region of the upper respiratory tract is separated from the trachea by the oropharynx and larynx. Although some studies treat the two regions as equivalent, we documented four differences between nasal and tracheal-bronchial submucosal glands of the pig.

*1)* Nasal glands had smaller maximal secretory rates and more densely distributed than tracheal glands (Figure 1). Nasal gland secretion rates were, on average, about 30% of the rates of tracheal glands but were ∼3 times more numerous. The predicted net result is that the volume of gland mucus secreted per a given unit of surface area should be equivalent in that region of the turbinates and in the trachea. For example, nasal gland secretion to 1 µM carbachol would be 29.5 glands/mm^2^×0.124 nl/min/gl = 3.66 nl/min for each mm^2^ of turbinate surface. Tracheal gland secretion would be 9×0.4 nl/min/gl = 3.6 nl/min for each mm^2^ of tracheal surface. One nanoliter of fluid produces 1 µm of depth over a 1 mm^2^ surface, so these numbers suggest that 2 min of secretion would produce ∼ 7 µm of fluid on either the nasal or tracheal surfaces—a value considered to be sufficient for normal mucociliary transport [51]. The situation in humans is the same. When glands in nose, rhinopharynx, pharynx, hypopharynx and trachea were compared the highest density occurred in the nose and the lowest in the trachea—but tracheal glands were much larger [52]. Within the cartilaginous airways, airway gland density is a positive linear function of airway lumen diameter across species [53], [54]; in 4–8 week old pigs, glands were not found in airways with an outer diameter smaller than 1 mm [55]. Almost the same relationship is found for gland size and airway diameter in human airways of different generations [56].

*2)* Pig nasal glands were almost unresponsive to Substance P (Figure 6, 7). This low-responsiveness was unexpected because SubP is a particularly potent and efficacious agonist for pig *tracheobronchial* submucosal glands [28], [43], [57], and because of evidence that it stimulates human nasal glands (see below). On the other hand, there is abundant evidence for regional differentiation within the respiratory epithelium e.g. [1], [58], [59], [60], [61].

*3)* Carbachol and forskolin were more potent for nasal than for tracheal glands. The carbachol and forskolin dose-response curves were left-shifted in nasal glands relative to tracheal glands (Figure 4).

These differences between turbinate and tracheobronchial glands may presage further differences among glands within the nasal cavity.

### Neonate vs. adult nasal glands

We expected that secretory responses of nasal glands to agonists would scale with gland size as animals grow. This was true for carbachol-stimulated secretion, which was ∼5-fold greater in adult than neonate glands (Figure 2DvH). By contrast, secretion rates to 3 µM forskolin, which are CFTR-dependent (Figure 3) and refs [26], [38], [40], [41], were ∼25-fold larger in adults, causing the ratio between forskolin and carbachol-stimulated nasal gland secretion to be 5-fold greater in adult than in neonate animals (Figure 4B, C). We do not know the basis for the increasing magnitude of CFTR-dependent secretion with age; it could be due to any combination of factors that increased NP_O_ of CFTR or basolateral Ca^2+^-activated K^+^ channels [50].

### Differences between human and pig nasal glands

Pig tracheal glands are more sensitive than human glands to SubP, [28], [57], but pig nasal glands are unresponsive to SubP (Figure 6, 7). What does SubP do to human nasal glands? Baraniuk and colleagues provide evidence that human nasal glands respond well to SubP [62]. They sprayed hypertonic saline into one nostril and collected lavage fluids from both nostrils. Only the sprayed nostril produced increased SubP, protein, lactoferrin, and mucoglycoprotein markers, suggesting glandular stimulation via local axon reflexes, consistent with abundant NK-1 receptor mRNA in the nasal glands [62], see also [63]. Together, the results suggest a four-way discordance in SubP sensitivity between pig and human nasal and tracheal glands. In humans, SubP sensitivity is high in nasal and low in tracheal glands, in pigs it is the reverse.

### Implications for pathogenesis in pigs and humans

Sinonasal disease has not yet been reported in CF pigs, but the CF piglet nasal epithelium has abnormal ion transport at birth [32] and we now show it also has deficient gland fluid secretion. In humans with CF, chronic rhinosinusitis (CRS) disease begins early and almost universally [see ref [64] and references therein]. It usually includes opacified sinuses, nasal polyps, and infections, and it differs in several ways from CRS in non-CF subjects [65]. The contribution of altered nasal gland secretion to human CF sinus disease is unknown, but increasing evidence suggests that it contributes to lung disease in CF patients [5], [8], [10], with sinonasal flora acting as a reservoir for pulmonary infection [2], [13].

In summary, these experiments reveal many features that distinguish nasal turbinate glands from tracheal glands. They also discover an unexpected increase in the relative role of forskolin-stimulated secretion in older pigs. In spite of these differences, fluid secretion from nasal glands in newborn or infant CFTR^-/-^ piglets is reduced to all mediators, (with some sparing in CFTR^ΔF508/ΔF508^ piglets), which will compound the previously demonstrated defects in tracheal glands (43). The nasal gland defects may compromise airway innate defenses at the earliest point of contact between mucosa and pathogens.

## Materials and Methods

### Pig nasal tissue

Piglets were offspring of CFTR^+/–^ or CFTR^+/ΔF508^ pigs made by homologous recombination in fibroblasts from outbred domestic pigs and subsequent somatic cell nuclear transfer [32]. Piglets were genotyped as described in references [34] and [33]. The ages and genotypes of piglets (n = 39) used for these experiments are shown in Table 1. All animal procedures were approved by the Institutional Animal Care and Use Committees of the University of Iowa, Iowa City, IA 52242. Nasal turbinate tissues (turbinate bone and overlying mucosa) were removed and shipped from the University of Iowa in cold physiological saline containing 10 mM glucose. Some nasal tissues purchased from Exemplar Genetics (Iowa City, IA) were delivered in the same manner. When possible, WT, Hz and CF samples from the same litter were co-shipped. The interval between euthanasia and experiments was 18–24 hrs. Nasal glands were also studied from 4 infant pigs (2 CF and 2 WT, 57–100 days old) and from 11 adult pigs (all WT, age >4 months). Adult tissues were obtained ∼ 1 hr postmortem (pentobarbital injection) following experiments carried out for other purposes. No WT adult pigs were euthanized specifically for these experiments; nevertheless, the experiments were approved by Stanford University Institutional Animal Care and Use Committee, Stanford, CA, 94305.

**Table 1:** Summary of subjects used.

	Genotype	Age (days)	n	Mean age	Total N
Neonate Control	+/+	1	8	2.3±0.7	12
	+/+	6	2		
	+/-	1	1		
	+/-	6	1		
Neonate CF	ΔF508/ΔF508	1	4	1.0±0.0	12
	-/ΔF508	1	1		
	-/-	1	7		
Infant Control	+/+	70	1	85±21	2
	+/+	100	1		
Infant CF	-/ΔF508	57	1	59±3	2
	-/ΔF508	61	1		
Adult Control	+/+	>120	11	Unknown	11

### Optical measurement of mucus secretion rates (mucus bubble method)

Secretion from individual glands was measured as described [66]. A piece of nasal turbinate mucosa ∼1 cm^2^ was dissected from the turbinate bone and mounted in a Sylgard-filled chamber with serosa in the bath (pH 7.4 and ∼290 mOsm Krebs-Ringer bicarbonate buffer containing glucose and 1 µM indomethacin to minimize prostaglandin release). The tissue surface was cleaned, dried, and about 10 µL of water-saturated mineral oil was layered onto its surface. Experiments were at 37°C; the tissue was superfused with warmed, humidified 95% O_2_–5% CO_2_. Pharmacological agents were diluted to final concentration with warmed, gassed bath solution and were added to the basolateral (serosal) side by complete bath replacement. Bubbles of secreted mucus were visualized within the oil layer by oblique illumination and digitally imaged with the macro lens of a camera. Images were measured using *ImageJ* software (http://rsb.info.nih.gov/ij/) and mucous volumes determined from the size of the spherical bubbles. Secretion rates were determined at 5 min intervals, these were sometimes plotted directly in addition to averaging rates over each experimental period (usually 30 min for each treatment). This method slightly underestimates the long-term rate of secretion to agonists like forskolin because it includes an initial period before secretion reaches maximum; and overestimates the rate to agonists like carbachol because it includes an initial rate of transiently high secretion.

Gland density was measured by counting the number of mucus bubbles after carbachol stimulation for 3 areas (totaling ∼3 mm^2^) randomly selected from within the region of maximal gland density (see Figure 1). The variation (standard error) of these three area of 1 mm^2^ were 2.8 (newborn nasal, n = 17), 1.6 (infant nasal, n = 4), and 0.6 (adult nasal, n = 7). Counts (glands/mm^2^) were averaged for 8 WT and 9 CF neonate piglets, 2 WT and 2 CF infant piglets, and 7 WT adult pigs.

### Histology

WT (n = 3) and CF (n = 3) neonate piglet and WT adult (n = 3) nasal turbinates were used for histological study. All tissues were fixed with 10% formaldehyde for 24 hours, dehydrated in an alcohol series and stained with hematoxylin and eosin and also with periodic acid-Schiff stain.

### Stimulation protocol

For most experiments we used a standard protocol of agonist addition designed to facilitate sequential testing of various mediators on the small amounts of tissues available. The standard protocol was: 1) a 20 min basal period, 2) 30 min of 3 µM forskolin, 3) 30 min of 0.1 µM carbachol + 3 µM Forskolin, and 4) 1 µM carbachol. The 3 µM forskolin dose was a compromise. It was chosen to be large enough to produce reproducible gland stimulation mediated by increased [cAMP]_i_ in control airways, but small enough (it is ∼ the EC_50_) to allow increases when combined with small concentrations of carbachol in a synergy paradigm. Carbachol at 0.1 µM is a near-threshold concentration for pig tracheal glands and so was considered to be appropriate for the synergy protocol, but as shown in the result section, 0.1 µM was determined to be suprathreshold for nasal glands. Carbachol at 1 µM elevated [Ca^2+^]_i_ sufficiently to recruit Ca^2+^-activated Cl^−^ channels (CaCCs) and produce glandular secretion that was at least partially CFTR-independent. A maximal concentration (∼ 3 µM) was avoided because the high level of secretion produced caused rapid merging of the adjacent bubbles of mucus. Merging is more of a problem in neonatal nasal tissue because of the high density of submucosal glands in that tissue (see Figure 2A–D).

### Reagents

Compounds (Sigma-Aldrich) were made fresh or maintained at −20°C as aliquots of stock solutions. Stock solutions of substance P (SubP), phosphoramidon (a protease inhibitor used together with substance P to prevent a potential breakdown of SubP), and carbachol were dissolved in sterile distilled water; indomethacin was in ethanol, and forskolin was in dimethyl sulfoxide (DMSO). Drugs in the gland secretion experiments were diluted 1:1,000 with bath solution immediately before use at the concentrations indicated. The highest DMSO concentration in our experiments was 0.1%.

### Statistics

Data are means±SEM, and Student's two tailed *t*-test for unpaired data was used to compare the means of different treatment groups unless otherwise indicated. The variations were tested with Levene's F-test and Mann-Whitney test was also used for unequal variations of groups. The difference between the two means was considered to be significant when *P*<0.05.

## Supporting Information

Figure S1 **Histology of nasal submucosal (superficial) glands from WT (upper panel) and CF (lower panel) neonatal pig nasal turbinates (hematoxylin and eosin with periodic acid-Schiff stain, x100).** Scale bar; 0.1 mm.
(TIF)
