# Supplementary figures and images for: Defective Fluid Secretion from Submucosal Glands of Nasal Turbinates from CFTR-/- and CFTRΔF508/ΔF508 Pigs
Source: PLoS One. 2011 Aug 31;6(8):e24424. doi: 10.1371/journal.pone.0024424 (PMC3164206; doi:10.1371/journal.pone.0024424)

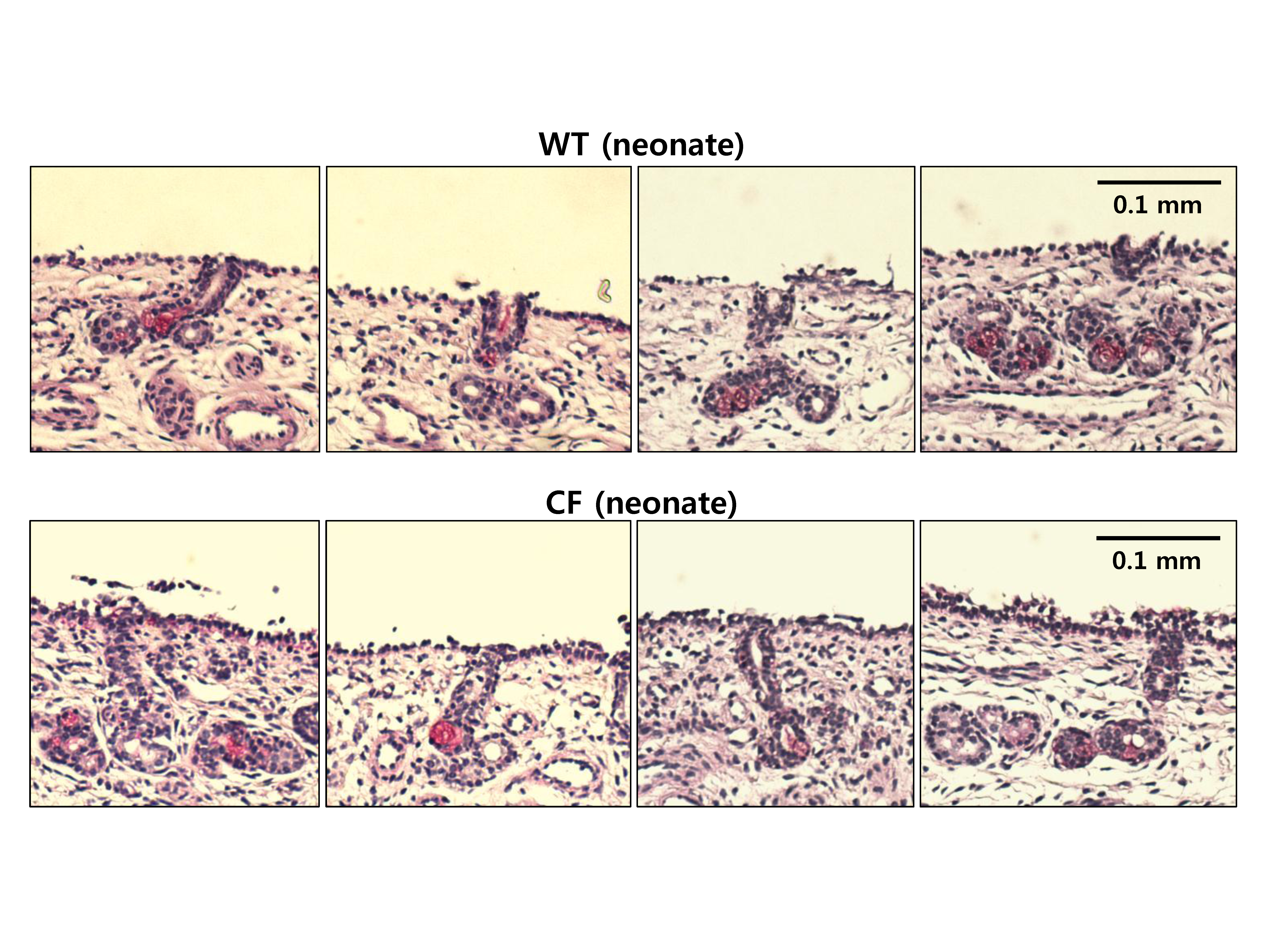

Supplement: Figure S1 — Histology of nasal submucosal (superficial) glands from WT (upper panel) and CF (lower panel) neonatal pig nasal turbinates (hematoxylin and eosin with periodic acid-Schiff stain, x100). Scale bar; 0.1 mm. (TIF) [file pone.0024424.s001.tif]
